# Environmental Regulation of 2-Acetyl-1-pyrroline Biosynthesis in Fragrant Rice: From Metabolic Pathways to Sustainable Quality Management

**DOI:** 10.3390/genes17030349

**Published:** 2026-03-22

**Authors:** Junjun Guo, Junyi Miao, Jin Chen, Deqian Huang, Chuyi Wang, Jiancheng Wen

**Affiliations:** 1 Rice Research Institute, Yunnan Agricultural University, Kunming 650201, China; 2024110035@stu.ynau.edu.cn (J.G.); 2025210242@stu.ynau.edu.cn (J.M.); cj15126398640@163.com (J.C.);; 2 Key Laboratory of Molecular Breeding for Dian-Type Japonica Hybrid Rice of Yunnan Education Department, Yunnan Agricultural University, Kunming 650201, China

**Keywords:** rice (*Oryza sativa* L.), 2-acetyl-1-pyrroline, environmental factors, aroma biosynthesis, volatile compounds

## Abstract

The market value of fragrant rice is largely defined by the presence and intensity of its aroma, which is primarily attributed to volatile compound 2-acetyl-1-pyrroline (2-AP). The biosynthesis of 2-AP is chiefly governed by recessive alleles of the *badh2* gene. Nevertheless, 2-AP accumulation is also profoundly shaped by environmental factors and agronomic management. Field practices—such as balanced nitrogen and potassium fertilization, supplementation with trace elements, and application of plant growth regulators like methyl jasmonate—promote 2-AP synthesis by increasing precursor availability and enhancing the activity of key enzymes. Additionally, tillage systems, alternate wetting and drying irrigation, optimal planting density, and harvest timing significantly affect aroma quality. Abiotic stresses, including moderate drought, salinity, optimal temperatures around 25 °C, and low light during grain filling, can also stimulate 2-AP accumulation, often through shifts in proline metabolism and activation of stress-responsive pathways involving GABA and methylglyoxal. Despite the promise of these strategies, several challenges persist, such as the common trade-off between yield and aroma intensity, complex genotype-by-environment interactions, and incomplete elucidation of the molecular mechanisms involved. Moving forward, integrating multi-omics analyses with smart agriculture technologies will be essential to unravel the regulatory networks underlying aroma formation and to advance the breeding of high-yielding fragrant rice varieties with stable aroma traits under changing climate scenarios.

## 1. Introduction

The production of high-quality rice has become a major focus of the global rice industry. Aroma, a key determinant of palatability and consumer acceptance, is among the most essential attributes defining rice quality [1,2,3,4]. Consumers show a strong preference for rice with a distinct and pleasant fragrance. Even subtle changes in aroma quality can significantly influence purchasing decisions. Because of its appealing scent and other superior traits, fragrant rice not only attracts more consumers but also often commands a premium price in the market. For decades, fragrant rice has maintained a prominent position in Asian markets, and has more recently gained increasing recognition and popularity in European and American markets as well [5,6]. Therefore, research into the formation and regulation of rice aroma is crucial for enhancing market competitiveness and promoting the development of agricultural economies.

A large number of volatile compounds contribute to rice aroma, with more than 300 compounds identified across different rice varieties [7,8,9,10,11]. However, 2-acetyl-1-pyrroline (2-AP) is widely recognized as the most important compound responsible for the characteristic popcorn-like aroma of fragrant rice. Owing to its extremely low odor threshold, even small changes in 2-AP concentration can significantly influence the sensory quality of rice [8,12,13]. Consequently, the level of 2-AP is commonly used as the primary indicator distinguishing aromatic from non-aromatic rice cultivars [14,15,16].

**Figure 1:**
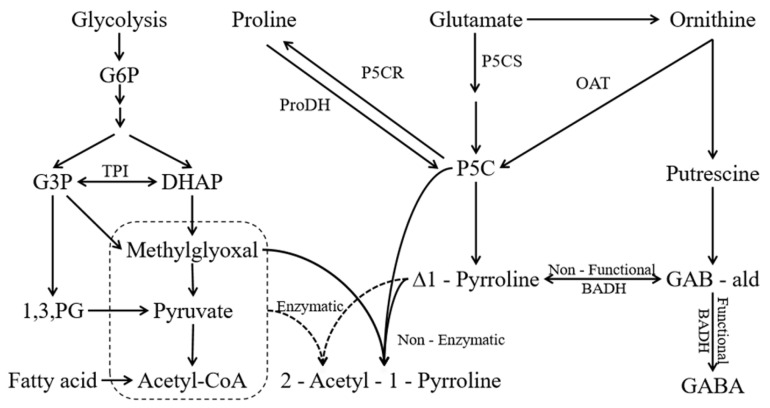
Biosynthetic pathway of 2-acetyl-1-pyrroline (2-AP) in rice. The diagram illustrates the major enzymatic and non-enzymatic routes leading to 2-AP formation. In non-aromatic rice, functional BADH2 converts GAB-ald to GABA, limiting 2-AP synthesis. In aromatic rice, loss of BADH2 function allows GAB-ald to be converted to Δ^1^-pyrroline, which reacts with methylglyoxal to form 2-AP. An alternative non-enzymatic pathway involves P5C derived from proline, ornithine, or glutamate directly reacting with methylglyoxal to produce 2-AP. Note: G6P: Glucose-6-phosphate; P5CS: Δ1-pyrrolidone-5-carboxylic acid synthase; P5CR: Pyrrolidone-5-carboxylic acid reductase; GAB-ald: γ-aminobutyraldehyde; GABA: γ-aminobutyric acid; ProDH: Proline dehydrogenase; BADH: Betaine aldehyde dehydrogenase; OAT: Ornithine aminotransferase; 1,3,PG: 1,3-diphosphoglycerate; DHAP: Dihydroxyacetone phosphate.

The biosynthesis of 2-AP in rice involves both enzymatic and non-enzymatic pathways (Figure 1). In non-aromatic rice, the functional BADH2 enzyme catalyzes the conversion of γ-aminobutyraldehyde (GAB-ald) to γ-aminobutyric acid (GABA), thereby preventing the formation of 2-AP. In contrast, aromatic rice varieties carry a loss-of-function mutation in the *badh2* gene, which leads to the accumulation of GAB-ald and its subsequent conversion into Δ^1^-pyrroline, a key precursor of 2-AP [17,18]. An additional non-enzymatic route involves the reaction between Δ^1^-pyrroline-5-carboxylate (P5C) and methylglyoxal, generating 2-AP under suitable conditions [19,20,21,22,23].

Although genetic factors play a central role in determining rice aroma, the accumulation of 2-AP is also strongly influenced by environmental conditions and agronomic management. Factors such as nutrient availability, irrigation regimes, temperature, light intensity, and abiotic stresses can affect precursor metabolism and enzyme activities involved in the 2-AP biosynthetic pathway. These interactions between genetic background and environmental regulation ultimately determine the level of aroma expressed in rice grains [17,24,25].

In this review, we summarize the current understanding of the genetic regulation, metabolic pathways, and environmental influences involved in 2-AP biosynthesis in rice. In particular, we highlight recent advances in plant physiological regulation and agronomic practices that affect aroma formation. By integrating these aspects, this review aims to provide a comprehensive overview of the mechanisms controlling rice aroma and to offer insights for improving the quality and stability of fragrant rice production.

## 2. Agronomic Management Strategies

**Figure 2:**
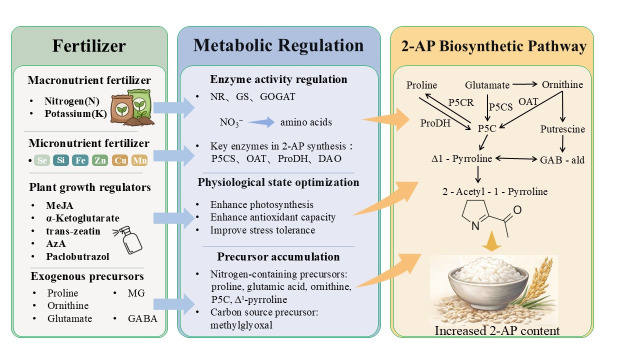
Mechanisms by Which Fertilizers Regulate 2-Acetyl-1-pyrroline Biosynthesis in Fragrant Rice. This figure summarizes how different nutrients influence 2-AP accumulation through coordinated effects on precursor supply and enzyme activities. Nitrogen fertilizer enhances nitrate reductase (NR), glutamine synthetase (GS), and glutamate synthase (GOGAT) activities, increasing glutamate and proline pools. Potassium acts as an enzyme activator to promote nitrogen assimilation. Trace elements such as selenium, zinc, and molybdenum serve as enzyme cofactors, upregulating genes including *OsP5CS*, *OsOAT*, and *OsProDH*, thereby increasing precursors (proline, P5C, Δ^1^-pyrroline) and promoting 2-AP synthesis. Plant growth regulators like methyl jasmonate further enhance photosynthetic efficiency and antioxidant capacity, indirectly supporting aroma formation.

While the genetic lesion in *badh2* is the prerequisite for aroma in fragrant rice, the ultimate intensity of 2-acetyl-1-pyrroline (2-AP) in the grain is not fixed. It is, instead, dynamically modulated by a range of agronomic practices that intervene in plant metabolism. These practices primarily function by altering the availability of key precursors (e.g., proline, P5C, methylglyoxal) and the activity of enzymes that lie outside the core BADH2 pathway, such as P5CS, OAT, and ProDH (Figure 2). This section critically evaluates how specific agronomic interventions—fertilization, cultivation methods, and precursor application—influence the 2-AP biosynthetic machinery, highlighting the underlying mechanisms and inherent trade-offs.

### 2.1. Reasonable Fertilizer Management

Nutrient management is the most direct and potent agronomic lever for influencing 2-AP accumulation. Its primary mechanism is to supply the carbon and nitrogen skeletons necessary for synthesizing amino acid precursors. Nitrogen (N), for instance, is fundamental. Rational N application increases the activity of key assimilatory enzymes like nitrate reductase (NR) and glutamine synthetase (GS), thereby expanding the pools of glutamate and proline—both direct precursors in the 2-AP pathway [26,27,28,29]. Potassium (K) acts synergistically by activating NR and facilitating nitrogen transport, ensuring that the increased nitrogen supply is effectively utilized for amino acid biosynthesis. [26,27,30].

Beyond these macronutrients, trace elements function as critical enzymatic cofactors, fine-tuning the metabolic flux towards 2-AP. For example, foliar selenium application has been shown to upregulate the transcription of *OsP5CS* and *OsOAT* while downregulating *Osbadh2*, leading to elevated activities of PRODH, P5CS, and OAT, and a consequent increase in Δ^1^-pyrroline and P5C levels [31,32,33]. Similarly, elements like zinc, manganese, and molybdenum are believed to enhance 2-AP synthesis through analogous effects on enzyme activity and gene expression, though their specific molecular targets are less well-defined [31,32,33,34,35,36,37,38,39,40].

Plant growth regulators also exhibit significant synergistic effects on 2-AP accumulation. Studies have shown that foliar spraying of exogenous substances such as methyl jasmonic acid (MeJA), α-ketoglutaric acid, trans-zeatin, and azelaic acid (AzA) can effectively increase 2-AP content in aromatic rice [41,42,43,44]. Their mechanisms of action include: increasing the content of photosynthetic pigments (such as chlorophyll a, chlorophyll b, and carotenoids) and net photosynthetic rate during the grain-filling period (e.g., paclobutrazol and methyl jasmonate); enhancing the activity of antioxidant enzymes (e.g., azelaic acid and methyl jasmonate); increasing the accumulation of 2-AP precursors (including Δ^1^-pyrroline, methylglyoxal, proline, and P5C); and enhancing the activity of key enzymes involved in 2-AP precursor synthesis (e.g., P5CS and OAT) (e.g., trans-zeatin, methyl jasmonate, and paclobutrazol) [41,42,43,44,45].

Notably, the effects of these diverse nutrients often converge on a common set of metabolic nodes. Whether it is nitrogen enhancing the substrate pool, or selenium boosting enzyme activity, the net result is an increase in the key intermediate P5C and its derivative Δ^1^-pyrroline. However, current research remains somewhat fragmented. Future research should focus on identifying the common regulatory targets of different nutrients in 2-AP biosynthesis; elucidating the fine-grained regulatory mechanisms of macro- and micronutrients in this metabolic pathway; and systematically analyzing the molecular interaction networks involved in aroma formation.

### 2.2. Farming Methods and Agronomic Management

Cultivation practices influence 2-AP accumulation not by directly supplying precursors, but by altering the plant’s overall physiological and metabolic status, often by inducing mild stress. Tillage practices, for example, affect soil properties and root growth. Compared to conventional rotary tillage, both plowing and no-tillage have been shown to increase grain 2-AP content [46,47]. This is associated with enhanced soil organic matter and microbial activity, which in turn promotes nitrogen uptake and upregulates OAT activity in the plant, leading to higher P5C and proline levels [46,47,48,49].

Irrigation management, particularly alternate wetting and drying (AWD), offers a clear example of stress-induced aroma enhancement. By introducing a controlled mild drought stress during the grain-filling period, AWD promotes proline accumulation as an osmoprotectant, thereby indirectly fueling the 2-AP biosynthetic pathway [50,51,52]. Similarly, high planting density can increase competition for light and resources, inducing a stress response that, up to a point, boosts precursor pools and enzyme activities [51]. Both strategies, however, walk a fine line. The mild stress that enhances aroma (e.g., an irrigation threshold of −25 cm) can, if not carefully managed, tip over into severe stress that reduces photosynthetic rate and grain weight [51]. This illustrates a central theme in fragrant rice production: a trade-off between yield and quality, where optimal aroma often resides at the boundary of plant stress tolerance.

Finally, harvest timing is critical because 2-AP is not a stable end-product. Its concentration in grains fluctuates during the ripening process, representing a dynamic balance between synthesis and degradation [53,54]. Delayed harvest can lead to a net loss of 2-AP as degradation outpaces synthesis, underscoring the need for precise timing to capture peak aroma [53].

In summary, while agricultural management practices can effectively modulate aroma biosynthesis in aromatic rice, the underlying molecular mechanisms remain to be fully elucidated. Key areas for future investigation include the impact of tillage methods on aroma-related metabolic pathways, the regulation of precursor accumulation by irrigation strategies, and the dynamic relationship between harvest time and the profile of aromatic volatiles.

### 2.3. Application of Exogenous Synthetic Precursors

A more direct strategy to enhance 2-AP content is to bypass potential metabolic bottlenecks by exogenously supplying the pathway’s building blocks. The logic is straightforward: if the plant has the genetic capacity to produce 2-AP (i.e., non-functional *badh2*), providing more precursors like proline, ornithine, or glutamate should drive the pathway forward [55,56,57]. These amino acids are readily converted into P5C, the direct precursor of Δ^1^-pyrroline [58]. Field trials have generally confirmed the efficacy of this approach, with foliar sprays of these compounds leading to significant increases in grain 2-AP.

Similarly, foliar application of γ-aminobutyric acid (GABA) during heading has been shown to increase both proline content and the activity of key enzymes like P5CS, further promoting 2-AP accumulation [59,60,61]. The effectiveness of GABA may stem from its dual role as both a potential precursor (via its link to the polyamine pathway) and a signaling molecule that can upregulate stress-responsive anabolic pathways [62,63]. The role of methylglyoxal (MG) is unique, as it provides the acetyl group for 2-AP. While MG is cytotoxic at high levels, its controlled increase through exogenous application or stress induction provides the necessary co-substrate to react with Δ^1^-pyrroline, particularly via the non-enzymatic pathway [21,22,64].

**Figure 3:**
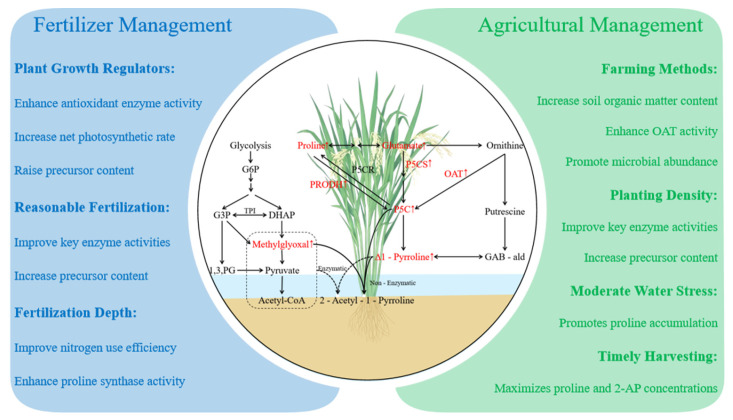
Cultivation strategies for enhanced 2-AP content in rice. The figure integrates multiple cultivation practices that modulate 2-AP biosynthesis. The figure integrates multiple cultivation practices that modulate 2-AP biosynthesis. These include fertilization, tillage, alternate wetting and drying (AWD) irrigation, planting density management, timely harvesting, and exogenous application of precursors/regulators. Each practice is labeled directly on the figure. These measures collectively increase precursor availability and key enzyme activities, enhancing aroma formation. Note: Key enzymes and metabolic intermediates in the 2-AP biosynthesis pathway that are targeted by these agronomic measures are highlighted in red.

In conclusion, field management practices exert a profound and complex influence on the biosynthesis and regulation of 2-AP. This complexity arises primarily because agronomic interventions alter the availability of metabolic precursors and the activity of key biosynthetic enzymes, thereby intricately modulating the final aroma content in rice grains (Figure 3). Future research should focus on three key aspects: the molecular regulatory mechanisms underlying amino acid-induced 2-AP biosynthesis, the dual role of the 2-AP biosynthesis pathway in stress responses, and the practical application of these metabolic networks for improving fragrant rice quality. These studies will provide a theoretical foundation for cultivating high-quality fragrant rice.

## 3. Environmental Stress Regulation

Environmental stresses often influence aroma formation through similar metabolic adjustments. Many stresses stimulate the accumulation of osmoprotectants such as proline and γ-aminobutyric acid (GABA), which are closely linked to the biosynthesis of 2-AP. In addition, stress-induced changes in carbohydrate metabolism may increase the production of methylglyoxal, providing additional substrates for non-enzymatic 2-AP formation. Therefore, different environmental factors may converge on common metabolic pathways regulating aroma accumulation in fragrant rice.

### 3.1. Drought Stress

Water availability is a primary environmental factor limiting rice productivity and modulating grain quality. In fragrant rice, drought stress has been extensively documented to influence the biosynthesis of 2-acetyl-1-pyrroline (2-AP), the key volatile compound responsible for its characteristic aroma [65,66,67,68]. Moderate drought stress during critical developmental stages, particularly heading and grain filling, has been shown to significantly elevate 2-AP content in grains [66,67].

The enhancement of aroma under water deficit conditions is closely tied to the plant’s physiological and metabolic stress responses. One of the most prominent adaptations is the accumulation of proline, a multifunctional osmoprotectant that stabilizes cellular structures, scavenges reactive oxygen species (ROS), and facilitates osmotic adjustment [68,69,70]. Proline serves as a direct precursor for 2-AP biosynthesis, as it can be converted into Δ^1^-pyrroline-5-carboxylate (P5C) and subsequently into Δ^1^-pyrroline—a key intermediate that reacts with methylglyoxal to form 2-AP [57,58].

Drought stress also modulates the activity of key enzymes in the proline metabolic pathway. The upregulation of Δ^1^-pyrroline-5-carboxylate synthetase (P5CS) and ornithine aminotransferase (OAT) enhances the conversion of glutamate and ornithine into P5C, thereby increasing the pool of precursors available for 2-AP synthesis [65,69]. Concurrently, changes in proline dehydrogenase (ProDH) activity help regulate proline turnover, ensuring a balance between stress protection and precursor availability [68].

At the molecular level, drought stress can influence the expression of aroma-related genes. In particular, the downregulation of *Osbadh2* under water deficit conditions reduces the conversion of γ-aminobutyraldehyde (GAB-ald) to γ-aminobutyric acid (GABA), thereby shunting GAB-ald toward Δ^1^-pyrroline formation and promoting 2-AP accumulation [18,24]. Additionally, drought stress may enhance the production of methylglyoxal (MG) through increased glycolytic flux, facilitating non-enzymatic 2-AP synthesis via its reaction with Δ^1^-pyrroline [21,64,70]. This dual enzymatic and non-enzymatic pathway highlights the complexity of 2-AP regulation under stress.

However, the outcome of drought stress on 2-AP accumulation is highly dependent on its intensity and duration. Mild to moderate stress during booting and grain filling optimizes aroma without severely compromising yield [66,67]. In contrast, severe or prolonged drought inhibits photosynthesis, disrupts nitrogen metabolism, and reduces the availability of amino acid precursors, ultimately diminishing both yield and grain quality [65,68]. Therefore, precise water management strategies, such as alternate wetting and drying (AWD) or regulated deficit irrigation (RDI), are recommended to induce mild stress during aroma-sensitive windows, thereby enhancing 2-AP content while sustaining productivity [50,51].

### 3.2. Salt Stress

Soil salinity is a growing concern in rice-producing regions and significantly impacts both plant growth and grain quality. In fragrant rice, salt stress has been shown to influence the accumulation of 2-AP, with moderate salinity often enhancing aroma while severe salinity impairs overall performance [71,72].

Salinity stress disrupts cellular ion homeostasis and induces osmotic stress, triggering the accumulation of compatible solutes such as proline and GABA [73,74]. Proline, in particular, accumulates under saline conditions and contributes to 2-AP biosynthesis by serving as a substrate for P5C and Δ^1^-pyrroline formation [58,74]. Increased proline levels are often accompanied by enhanced activity of P5CS and OAT, which facilitate the conversion of glutamate and ornithine into P5C [56,57].

Salt stress also promotes glycolysis and the accumulation of methylglyoxal, a reactive carbonyl compound that participates in the non-enzymatic formation of 2-AP [64,70]. The combination of elevated precursor pools and enhanced MG production under moderate salinity may synergistically promote 2-AP synthesis. Furthermore, salt stress modulates the expression of genes involved in aroma biosynthesis. The *Osbadh2* gene, responsible for BADH2 activity, may be downregulated under saline conditions, reducing the conversion of GAB-ald to GABA and thereby favoring 2-AP accumulation [24,74].

However, the relationship between salinity and aroma is dose-dependent. While low to moderate salinity can enhance 2-AP content, high salinity severely inhibits photosynthesis, reduces tillering, and impairs grain filling, leading to yield losses and inconsistent aroma quality [71,75]. Therefore, optimizing salt stress levels through soil management or the use of salt-tolerant varieties is essential for balancing aroma enhancement with productivity.

Soil salinity significantly affects 2-AP accumulation in rice. Both the aroma-related gene *Osbadh2* and its homolog *Osbadh1* are associated with salt tolerance in rice [73,74,75]. Current understanding suggests that *Osbadh1* primarily mediates salt tolerance during early seedling stages, while *Osbadh2* contributes to salt tolerance in later developmental phases [24]. Interestingly, fragrant rice cultivated under saline conditions exhibits elevated 2-AP content, which shows a significant positive correlation with proline accumulation but not with GABA levels [71]. While moderate salt stress enhances 2-AP biosynthesis in fragrant rice, further investigation is required to elucidate the molecular mechanisms underlying salinity-regulated 2-AP synthesis to minimize potential yield penalties.

### 3.3. Temperature

Temperature is a critical environmental factor governing metabolic processes and volatile compound formation in plants. In fragrant rice, temperature during heading and grain filling plays a decisive role in determining 2-AP accumulation [76,77].

Optimal temperatures around 25 °C have been associated with the highest 2-AP content in grains [76,77]. Under such conditions, amino acid and polyamine metabolism—particularly involving glutamate, proline, and ornithine—is actively channeled toward the formation of Δ^1^-pyrroline, a key intermediate in 2-AP biosynthesis [72,78]. Enzymes such as P5CS and OAT exhibit enhanced activity at moderate temperatures, promoting the conversion of precursors into P5C and subsequently into Δ^1^-pyrroline [72,77].

Temperature also affects the stability and degradation of 2-AP. While high temperatures (e.g., 32/26 °C) may initially stimulate synthesis, they also accelerate the volatility and degradation of 2-AP, resulting in lower net accumulation [76,79]. Conversely, cooler temperatures (e.g., 22/16 °C) may slow degradation rates, allowing for sustained accumulation, although they may also limit precursor supply due to reduced metabolic activity [77,79].

At the molecular level, temperature fluctuations can modulate the expression of aroma-related genes. Heat stress may upregulate stress-responsive transcription factors that indirectly influence the proline and GABA pathways, while cold stress may alter membrane fluidity and compartmentalization of metabolic intermediates [80,81]. However, the precise regulatory networks linking temperature sensing to 2-AP biosynthesis remain poorly understood and warrant further investigation.

Given the increasing frequency of temperature extremes under climate change, understanding the thermal regulation of 2-AP biosynthesis is crucial for developing resilient fragrant rice varieties and optimizing planting schedules to preserve aroma quality [76,82].

### 3.4. Light Intensity

Light is an essential regulator of plant growth, photosynthesis, and secondary metabolism. In fragrant rice, light intensity during grain filling has been shown to influence 2-AP accumulation, although the mechanisms remain less explored compared to other abiotic factors [83,84,85,86].

Under optimal light conditions, enhanced photosynthetic activity increases the supply of carbon skeletons and energy required for amino acid and polyamine metabolism, thereby supporting the synthesis of 2-AP precursors such as proline and ornithine [83,85]. Increased carbohydrate metabolism may also promote glycolytic flux, generating methylglyoxal that can participate in non-enzymatic 2-AP formation [70,86].

Conversely, low light intensity or shading during grain filling has been reported to increase 2-AP content in some studies [83]. This seemingly paradoxical effect may be attributed to shifts in nitrogen metabolism and stress signaling under light limitation. Shading can induce the accumulation of proline and GABA as part of a stress response, which may in turn enhance precursor availability for 2-AP synthesis [83,84]. However, low light also reduces photosynthetic efficiency and biomass accumulation, often leading to yield penalties [83,86].

The spectral composition of light also matters. Supplemental lighting with red or blue spectra during grain filling may improve yield but has been shown to reduce 2-AP content compared to natural light, possibly due to altered expression of genes involved in proline and GABA metabolism [84,85]. These findings suggest that light quality and intensity interact with other environmental factors—such as water and temperature—to modulate aroma formation [86].

Future research should focus on elucidating the molecular mechanisms underlying light-regulated 2-AP biosynthesis, including the role of photoreceptors, circadian clocks, and light-responsive transcription factors in controlling flux through the proline–P5C–2-AP pathway.

### 3.5. Biotic Stress

Biotic stresses caused by pathogens and insect pests are major constraints to rice production, but their effects on grain aroma remain poorly understood. Although no direct evidence has conclusively demonstrated that pest or disease infestation increases 2-AP content, several lines of evidence suggest indirect links through stress-induced metabolic adjustments [87,88,89].

When plants are attacked by pathogens or herbivores, they activate defense responses involving the synthesis of secondary metabolites, ROS scavenging systems, and stress-related signaling molecules. Among these, proline and GABA often accumulate as part of the general stress response [87,88]. Proline contributes to cellular redox balance and may also serve as a precursor for 2-AP biosynthesis via the P5C–Δ^1^-pyrroline route [58]. GABA, which accumulates under biotic stress, is synthesized from glutamate via glutamate decarboxylase (GAD) and is closely linked to the polyamine degradation pathway—both of which intersect with 2-AP metabolism [89,90].

In addition, pathogen infection may alter primary metabolism, including glycolysis and the TCA cycle, potentially increasing the availability of methylglyoxal for non-enzymatic 2-AP formation [70]. However, severe biotic stress typically leads to reduced photosynthetic capacity, impaired grain filling, and decreased yield, which may offset any potential gains in aroma quality [87,89].

The interaction between biotic and abiotic stresses adds further complexity. For example, drought- or salinity-stressed plants may be more susceptible to certain pathogens, and the combined stress responses may synergistically or antagonistically affect 2-AP accumulation [82]. Understanding these interactions is essential for integrated pest and nutrient management strategies that aim to preserve both yield and aroma quality.

Future research should employ transcriptomic and metabolomic approaches to dissect the metabolic crosstalk between defense pathways and aroma biosynthesis, and to identify candidate genes or metabolites that could serve as targets for breeding or agronomic intervention.

### 3.6. Stress-Induced Metabolic Regulation

Rice plants have evolved diverse protective mechanisms under stress conditions, involving the biosynthesis of signaling molecules and protective compounds including GABA, proline, methylglyoxal, and calmodulin [63,70,91,92]. GABA production occurs through both the polyamine degradation pathway and the GABA shunt pathway (mediated by *OsGAD* gene activity), with its stress-responsive functions being achieved through either *Osbadh2* gene activation or GABA shunt activity [90,93,94]. Cellular perception of stress intensity and nature is mediated by fluctuations in Ca^2+^ and/or H^+^ concentrations, which subsequently induce *OsGAD* gene expression and GABA production via the GABA shunt pathway [93,94]. The binding of GABA to its receptors triggers Ca^2+^ release from intracellular stores, elevating cytosolic Ca^2+^ levels. This Ca^2+^ surge amplifies the formation of Ca^2+^/calmodulin (CaM) complexes and enhances stress signaling cascades, ultimately activating stress-responsive genes [62]. Research indicates that the GABA shunt pathway exhibits heightened activity under stress conditions, with GABA receptors potentially regulating mineral uptake to modulate enzymes in stress-related metabolic pathways.

**Figure 4:**
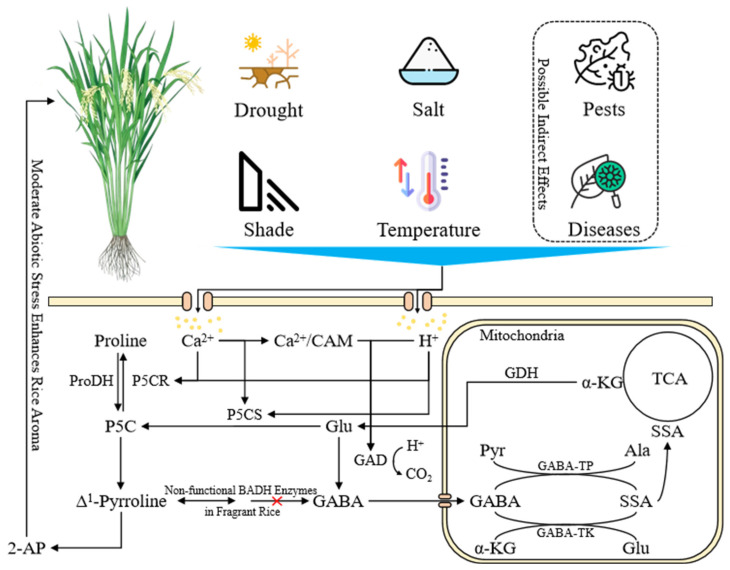
Environmental stress-induced 2-AP accumulation in aromatic rice. Environmental stresses such as drought, salinity, and other adverse conditions can stimulate the accumulation of 2-AP through stress-induced metabolic pathways. Stress signals trigger the influx of Ca^2+^ and/or H^+^ ions, activating genes such as *OsP5CS* and *OsGAD*, which participate in proline biosynthesis and the γ-aminobutyric acid (GABA) shunt pathway. These processes lead to the production of important intermediates including Δ^1^-pyrroline-5-carboxylate (P5C) and Δ^1^-pyrroline, which are essential precursors for 2-AP formation. Arrows indicate metabolic pathways and regulatory interactions involved in stress-induced aroma formation. Note: P5CR: Δ^1^-Pyrroline-5-Carboxylate Reductase; P5CS: Δ^1^-pyrrolidone-5-carboxylic acid synthase; ProDH: Proline Dehydrogenase; GABA: γ-aminobutyric acid; BADH: Betaine aldehyde dehydrogenase; Glu: Glutamic; P5C: Δ1-Pyrrolidone-5-carboxylic acid; α-KG: α-Ketoglutaric acid; Pyr: Pyruvic acid; Ala: Alanine; SSA: Succinic semialdehyde; TCA: tricarboxylic acid cycle; GDH: Glutamic Acid Decarboxylase.

An additional mechanism for stress-induced 2-AP accumulation involves the concurrent release of both starch-bound and free 2-AP pools [24]. Figure 4 illustrates the molecular framework underlying fragrant rice’s environmental stress adaptation. Stress stimuli initiate Ca^2+^ and/or H^+^ influx, activating *OsP5CS* and *OsGAD* genes whose protein products regulate signal transduction through the mitogen-activated protein kinase pathway [80]. These genetic responses lead to: (1) proline biosynthesis via polyamine degradation, and (2) GABA production through the GABA shunt pathway—both serving as critical signaling molecules for stress tolerance enhancement. Concurrently, stress conditions stimulate methylglyoxal generation through glycolysis and elevate polyamine concentrations. While methylglyoxal functions as a housekeeping signaling molecule at physiological concentrations, it exhibits cytotoxicity at elevated levels [92]. The stress-induced methylglyoxal surplus can be either enzymatically converted to pyruvate or non-enzymatically react with Δ^1^-pyrroline (an aromatic compound intermediate) to form 2-AP [21].

Taken together, environmental parameters including soil properties, temperature, relative humidity, moisture content, and pH significantly influence both aroma quality and plant survival in fragrant rice from flowering through maturity, primarily through modulation of glycolysis, 2-AP biosynthesis, and GABA shunt pathways. Climate change, particularly during critical growth stages, now represents a substantial threat to aroma quality in major rice-producing regions. This underscores the urgent need for comprehensive research to elucidate the genetic regulation, molecular mechanisms, and biochemical pathways governing environmentally responsive 2-AP synthesis.

## 4. Prospects and Challenges

Numerous studies have established significant correlations between 2-AP accumulation in aromatic rice and various mineral nutrients (e.g., nitrogen, potassium, selenium, molybdenum) as well as environmental factors (e.g., drought, salt stress, temperature fluctuations) [16,24,58,95]. The regulatory influence of these factors on 2-AP synthesis is closely associated with the metabolic dynamics of precursor amino acids like proline, and the expression and activity of key enzymes encoded by genes such as *Osbadh2*, *OsP5CS*, and *OsOAT*. However, the specific signaling pathways and detailed molecular mechanisms remain elusive. A critical aspect is that proline and related compounds serve dual roles as osmoprotectants and direct precursors for 2-AP biosynthesis [57,91]. Understanding how their metabolism and partitioning are regulated under specific environmental conditions constitutes a central question demanding further investigation. Systematically elucidating the entire regulatory cascade—from environmental perception and signal transduction to genetic regulation and phenotypic output—is essential to unravel the mechanisms of aroma formation.

The interactions among environmental factors are highly complex. Under field conditions, high temperature and drought often co-occur, and nutrient management is frequently intertwined with water regimes [82]. These factors can exert synergistic, additive, or antagonistic effects on 2-AP synthesis. Most current studies, however, were based on single-factor experiments, lacking comprehensive analyses of these interactions. For instance, while foliar application of proline enhances 2-AP content under optimal hydration, its efficacy may be diminished or reversed under severe drought stress [57]. Deciphering these complex interactions is vital for developing precision agronomic strategies adaptable to climate-variable environments and provides a theoretical foundation for mitigating aroma instability in the face of climate change.

Furthermore, aroma expression is strongly influenced by genotype-by-environment (G × E) interactions. A variety exhibiting high aroma in one region may show significantly reduced levels when grown in a different environment [96]. This G × E effect poses a major challenge to the consistent quality and regional promotion of aromatic rice varieties. Currently, evaluation systems for assessing the aroma stability of diverse germplasm across environments are inadequate, and molecular markers predictive of such stability for breeding applications are scarce. Therefore, identifying superior allelic variants or gene combinations that confer stable, high aroma expression across diverse environments is a crucial goal for future genetic improvement.

Central to these challenges is the pervasive trade-off between aroma and yield. Moderate drought stress can boost 2-AP accumulation but often at the cost of reduced seed set and grain weight [66]. Similarly, shading may promote aroma synthesis but limits photosynthetic accumulation [83]. Genetic interventions might disrupt carbon–nitrogen homeostasis, impairing normal growth [97]. Consequently, future research must prioritize “win-win” strategies that maximize aroma pathway activation without significantly compromising yield, thereby overcoming the traditional yield–quality dilemma.

Addressing these challenges requires an integrated, multidisciplinary approach. Integrating systems biology approaches—including transcriptomics, metabolomics, and proteomics—will help reconstruct gene regulatory networks and metabolic models centered on 2-AP biosynthesis [81]. This will facilitate the identification of key transcription factors, signaling molecules, and novel regulators (e.g., non-coding RNAs). Concurrently, leveraging technologies like IoT sensors, drone remote sensing, and AI algorithms can enable data-driven, smart agronomic management. Such tools allow for variable-rate input application and the precise implementation of mild stress within critical windows, optimizing aroma while minimizing yield penalty [98,99,100]. In genetic improvement, large-scale resequencing and phenotyping of global germplasm resources, coupled with genome-wide association studies, can identify alleles conferring aroma stability and environmental adaptability [101,102,103,104]. Genome editing technologies like CRISPR-Cas9 offer precise means to tailor key genes, accelerating the development of novel varieties with high, stable aroma and yield [105,106].

Currently, research on the environmental regulation of 2-AP biosynthesis is evolving from phenomenological description towards mechanistic understanding and precise modulation. The challenges are multifaceted yet present significant opportunities for innovation. By integrating multidisciplinary expertise from molecular biology, crop physiology, bioinformatics, smart agriculture, and food engineering, innovative research can be conducted along the entire chain—from molecules to populations, and from pre- to post-harvest management. Deciphering the environment–aroma interaction code will enable the cultivation of superior varieties and the development of scientific cultivation techniques. This integrated approach is key to producing aromatic rice with rich and stable aroma, high yield, and superior quality, ultimately realizing full-chain aroma quality management from field to table and meeting the growing global demand for premium rice products.

## Data Availability

No new data were created or analyzed in this study.

## References

[1] Prodhan Z.H., Samonte S.O.P.B., Sanchez D.L., Talukder S.K. (2024). Profiling and Improvement of Grain Quality Traits for Consumer Preferable Basmati Rice in the United States. Plants.

[2] Bhattacharya K.R. (2011). Rice Quality: A Guide to Rice Properties and Analysis.

[3] Fang P., Zhou Z., Wang H., Zhang L. (2024). Consumer Preference and Willingness to Pay for Rice Attributes in China: Results of a Choice Experiment. Foods.

[4] Sattari A., Mahdinezhad N., Fakheri B., Noroozi M., Beheshtizadeh H. (2015). Improvement of the Eating and Cooking Qualities of Rice: A Review. Int. J. Farming Allied Sci..

[5] Bairagi S., Demont M., Custodio M.C., Ynion J. (2020). What Drives Consumer Demand for Rice Fragrance? Evidence from South and Southeast Asia. Br. Food J..

[6] Giraud G. (2013). The World Market of Fragrant Rice, Main Issues and Perspectives. Int. Food Agribus. Manag. Rev..

[7] Lu L., Hu Z., Fang C., Hu X. (2023). Characteristic Flavor Compounds and Functional Components of Fragrant Rice with Different Flavor Types. Foods.

[8] Widjaja R., Craske J.D., Wootton M. (1996). Comparative Studies on Volatile Components of Non-fragrant and Fragrant Rices. J. Sci. Food Agric..

[9] Jia Q., Ma Y., Bi W., Su D. (2025). Analysis of Volatile Organic Compounds in Ten Types of Tribute Rice Based on Headspace-Gas Chromatography-Ion Mobility Spectrometry Technology. Food Chem. X.

[10] Chen J., Liu Y., Yang M., Shi X., Mei Y., Li J., Yang C., Pu S., Wen J. (2023). Analysis of the Differences in Volatile Organic Compounds in Different Rice Varieties Based on GC-IMS Technology Combined with Multivariate Statistical Modelling. Molecules.

[11] Xiong Y., Zheng X., Tian X., Wang C., Chen J., Zhou L., Xu D., Wang J., Gilard V., Wu M. (2024). Comparative Study of Volatile Organic Compound Profiles in Aromatic and Non-Aromatic Rice Cultivars Using HS-GC–IMS and Their Correlation with Sensory Evaluation. LWT.

[12] Wei X., Sun Q., Methven L., Elmore J.S. (2021). Comparison of the Sensory Properties of Fragrant and Non-Fragrant Rice (*Oryza sativa*), Focusing on the Role of the Popcorn-like Aroma Compound 2-Acetyl-1-Pyrroline. Food Chem..

[13] Lorieux M., Petrov M., Huang N., Guiderdoni E., Ghesquière A. (1996). Aroma in Rice: Genetic Analysis of a Quantitative Trait. Theor. Appl. Genet..

[14] Bhattacharjee P., Singhal R.S., Kulkarni P.R. (2002). Basmati Rice: A Review. Int. J. Food Sci. Technol..

[15] Singh S.P., Singh M.K., Kumar S., Sravan U.S. (2019). Cultivation of Aromatic Rice: A Review. Agronomic Crops.

[16] Champagne E.T. (2008). Rice Aroma and Flavor: A Literature Review. Cereal Chem..

[17] Nadaf A.B., Wakte K.V., Zanan R.L. (2014). 2-Acetyl-1-Pyrroline Biosynthesis: From Fragrance to a Rare Metabolic Disease. J. Plant Sci. Res..

[18] Bradbury L.M.T., Fitzgerald T.L., Henry R.J., Jin Q., Waters D.L.E. (2005). The Gene for Fragrance in Rice. Plant Biotechnol. J..

[19] Romanczyk L.J., McClelland C.A., Post L.S., Aitken W.M. (1995). Formation of 2-Acetyl-1-Pyrroline by Several Bacillus Cereus Strains Isolated from Cocoa Fermentation Boxes. J. Agric. Food Chem..

[20] Efferson J.N. (1985). Rice Quality in World Markets. IRRI Reports Rice Grain Quality and Marketing.

[21] Huang T.-C., Teng C.-S., Chang J.-L., Chuang H.-S., Ho C.-T., Wu M.-L. (2008). Biosynthetic Mechanism of 2-Acetyl-1-Pyrroline and Its Relationship with Δ1-Pyrroline-5-Carboxylic Acid and Methylglyoxal in Aromatic Rice (*Oryza sativa* L.) Callus. J. Agric. Food Chem..

[22] Wu M., Chou K., Wu C., Chen J., Huang T. (2009). Characterization and the Possible Formation Mechanism of 2-acetyl-1-pyrroline in Aromatic Vegetable Soybean (*Glycine max* L.). J. Food Sci..

[23] Huang Y., Huang L., Cheng M., Li C., Zhou X., Ullah A., Sarfraz S., Khatab A., Xie G. (2024). Progresses in Biosynthesis Pathway, Regulation Mechanism and Potential Application of 2-Acetyl-1-Pyrroline in Fragrant Rice. Plant Physiol. Biochem..

[24] Prodhan Z.H., Qingyao S. (2020). Rice Aroma: A Natural Gift Comes with Price and the Way Forward. Rice Sci..

[25] Roy S., Banerjee A., Basak N., Kumar J., Mandal N.P. (2020). Aromatic Rice. The Future of Rice Demand: Quality Beyond Productivity.

[26] Deng S., Ashraf U., Nawaz M., Abbas G., Tang X., Mo Z. (2022). Water and Nitrogen Management at the Booting Stage Affects Yield, Grain Quality, Nutrient Uptake, and Use Efficiency of Fragrant Rice under the Agro-Climatic Conditions of South China. Front. Plant Sci..

[27] Dou Z., Tang S., Li G., Liu Z., Ding C., Chen L., Wang S., Ding Y. (2017). Application of Nitrogen Fertilizer at Heading Stage Improves Rice Quality under Elevated Temperature during Grain-filling Stage. Crop. Sci..

[28] Yu Q.-G., Ye J., Yang S.-N., Fu J.-R., Ma J.-W., Sun W.-C., Jiang L.-N., Wang Q., Wang J.-M. (2013). Effects of Nitrogen Application Level on Rice Nutrient Uptake and Ammonia Volatilization. Rice Sci..

[29] Wu W., Wang Y., Wang L., Xu H., Zörb C., Geilfus C.-M., Xue C., Sun Z., Ma W. (2022). Booting Stage Is the Key Timing for Split Nitrogen Application in Improving Grain Yield and Quality of Wheat–A Global Meta-Analysis. Field Crops Res..

[30] Luo Y., Xiao L., Pan S., Nie J., Li Y., Tang X. (2014). Effects of Potassium Fertilizer on Aroma and Quality of Aromatic Rice. Southwest China J. Agric. Sci..

[31] Luo H., He L., Du B., Pan S., Mo Z., Duan M., Tian H., Tang X. (2020). Biofortification with Chelating Selenium in Fragrant Rice: Effects on Photosynthetic Rates, Aroma, Grain Quality and Yield Formation. Field Crops Res..

[32] Luo H., He L., Lai R., Liu J., Xing P., Tang X. (2021). Selenium Applications Enhance 2-acetyl-1-pyrroline Biosynthesis and Yield Formation of Fragrant Rice. Agron. J..

[33] Wang R., Zhang M., Chen T., Shen W., Dai J., Zhang H., Zhang H. (2025). Enhanced Leaf Photosynthesis, Grain Yield, Rice Quality and Aroma Characteristics in Rice Grains (*Oryza sativa* L.) with Foliar Application of Selenium Nanoparticles. Plant Physiol. Biochem..

[34] Chan-In P., Jamjod S., Prom-U-Thai C., Rerkasem B., Russell J., Pusadee T. (2024). Application of Silicon Influencing Grain Yield and Some Grain Quality Features in Thai Fragrant Rice. Plants.

[35] Ghasal P.C., Shivay Y.S., Pooniya V., Kumar P., Verma R.K. (2016). Zinc Fertilization Enhances Growth and Quality Parameters of Aromatic Rice (*Oryza sativa* L.) Varieties. Indian J. Plant Physiol..

[36] Inpradit W., Jamjod S., Prom-U-Thai C., Pusadee T. (2023). Genotypic Variation in Thai Fragrant Rice in Response to Manganese Application and Its Effects on 2-Acetyl-1-Pyrroline Content, Productivity and Gene Expression. Agronomy.

[37] Li M., Ashraf U., Tian H., Mo Z., Pan S., Anjum S.A., Duan M., Tang X. (2016). Manganese-Induced Regulations in Growth, Yield Formation, Quality Characters, Rice Aroma and Enzyme Involved in 2-Acetyl-1-Pyrroline Biosynthesis in Fragrant Rice. Plant Physiol. Biochem..

[38] Mo Z., Lei S., Ashraf U., Khan I., Li Y., Pan S., Duan M., Tian H., Tang X. (2017). Silicon Fertilization Modulates 2-Acetyl-1-Pyrroline Content, Yield Formation and Grain Quality of Aromatic Rice. J. Cereal Sci..

[39] Mo Z., Liu Q., Xie W., Ashraf U., Abrar M., Pan S., Duan M., Tian H., Wang S., Tang X. (2020). Ultrasonic Seed Treatment and Cu Application Modulate Photosynthesis, Grain Quality, and Cu Concentrations in Aromatic Rice. Photosynthetica.

[40] Imran M., Hussain S., He L., Ashraf M.F., Ihtisham M., Warraich E.A., Tang X. (2021). Molybdenum-Induced Regulation of Antioxidant Defense-Mitigated Cadmium Stress in Aromatic Rice and Improved Crop Growth, Yield, and Quality Traits. Antioxidants.

[41] Zhang Y., Ren Y., Yang D., Liu H., Zhang Y., Wang X., Bai F., Cheng S. (2023). Foliar Methyl Jasmonate (MeJA) Application Increased 2-Acetyl-1-Pyrroline (2-AP) Content and Modulated Antioxidant Attributes and Yield Formation in Fragrant Rice. J. Plant Physiol..

[42] Li Q., Hu H., Wang X., Liu F., Sun M., Ren Y., Qiu X. (2025). Effects of Exogenous Azelaic Acid (Aza) Application on 2-Acetyl-1-Pyrroline (2ap) Content, the Antioxidant Defense System, and Yield in Fragrant Rice. J. Plant Growth Regul..

[43] Fu X., Gui R., Li W., Gao Z., Ashraf U., Tan J., Ye Q., Chen J., Xie H., Mo Z. (2021). Nitrogen and α-Ketoglutaric Acid Application Modulate Grain Yield, Aroma, Nutrient Uptake and Physiological Attributes in Fragrant Rice. J. Plant Growth Regul..

[44] Xing P., Luo H., He Z., He L., Zhao H., Tang X., Duan M. (2023). Trans-Zeatin Induced Regulation of the Biosynthesis of 2-Acetyl-1-Pyrroline in Fragrant Rice (*Oryza sativa* L.) Seedlings. BMC Plant Biol..

[45] Hu F., Lu J., Zhai L., Qiu X., Du B., Xu J. (2025). Effects of Foliar Application of Paclobutrazol on Grain Yield, Aroma, and Canopy Radiation Use Efficiency of Aromatic Rice. Biology.

[46] Ren Y., Cheng S., Pan S., Tian H., Duan M., Wang S., Tang X. (2021). Effect of Conservation Tillage Practices on Aroma, Yield and Quality of Mechanical-Transplanting Fragrant Rice. J. Plant Interact..

[47] Zhao R., Luo H., Wang Z., Hu L. (2020). Benefits of Continuous Plow Tillage to Fragrant Rice Performance. Agron. J..

[48] Du B., He L., Lai R., Luo H., Zhang T., Tang X. (2020). Fragrant Rice Performances in Response to Continuous Zero-Tillage in Machine-Transplanted Double-Cropped Rice System. Sci. Rep..

[49] Du P., Luo H., He J., Mao T., Du B., Hu L. (2019). Different Tillage Induces Regulation in 2-Acetyl-1-Pyrroline Biosynthesis in Direct-Seeded Fragrant Rice. BMC Plant Biol..

[50] Ashraf U., Hussain S., Naveed Shahid M., Anjum S.A., Kondo M., Mo Z., Tang X. (2022). Alternate Wetting and Drying Modulated Physio-biochemical Attributes, Grain Yield, Quality, and Aroma Volatile in Fragrant Rice. Physiol. Plant..

[51] Wang W.-X., Jiang S.-C., Xing D.-Y., Du B. (2022). Effect of Planting Density and Irrigation Management on the Growth, Yield, and 2-Acetyl-△ 1-Pyrroline Content of Fragrant Rice. J. Soil Sci. Plant Nutr..

[52] Sriphirom P., Rossopa B. (2024). Greenhouse Gas Mitigation and Yield Production of Thai Fragrant Rice Cultivation under Alternate Wetting and Drying Water Management. Proceedings of the IOP Conference Series: Earth and Environmental Science.

[53] Zhang J., Tong T., Potcho P.M., Li L., Huang S., Yan Q., Tang X. (2021). Harvest Time Effects on Yield, Quality and Aroma of Fragrant Rice. J. Plant Growth Regul..

[54] Chakraborty R., Roy T.S., Sakagami J.-I. (2024). Impact of Harvesting Time on Grain Yield, Physicochemical Attributes, and 2-Acetyl-1-Pyrroline Biosynthesis in Aromatic Rice. Agronomy.

[55] Huang S., Deng Q., Zhao Y., Chen G., Geng A., Wang X. (2023). L-Glutamate Seed Priming Enhances 2-Acetyl-1-Pyrroline Formation in Fragrant Rice Seedlings in Response to Arsenite Stress. J. Agric. Food Chem..

[56] Renuka N., Barvkar V.T., Ansari Z., Zhao C., Wang C., Zhang Y., Nadaf A.B. (2022). Co-Functioning of 2AP Precursor Amino Acids Enhances 2-Acetyl-1-Pyrroline under Salt Stress in Aromatic Rice (*Oryza sativa* L.) Cultivars. Sci. Rep..

[57] Luo H., Zhang T., Zheng A., He L., Lai R., Liu J., Xing P., Tang X. (2020). Exogenous Proline Induces Regulation in 2-Acetyl-1-Pyrroline (2-AP) Biosynthesis and Quality Characters in Fragrant Rice (*Oryza sativa* L.). Sci. Rep..

[58] Yin S., Mu X., Jiang R., Liu Y., Sun B., Tian H., Liang S. (2024). Exploring Synthetic Pathways for 2-Acetyl-1-Pyrroline: Challenges and Prospects. Food Rev. Int..

[59] Pan Y., Chen Y., Wang C., Li H., Huang D., Zhou D., Wang Z., Zhao L., Gong R., Zhou S. (2021). Metabolism of γ-Aminobutyrate and 2-Acetyl-1-Pyrroline Analyses at Various Grain Developmental Stages in Rice (*Oryza sativa* L.). Chin. J. Rice Sci..

[60] Gao Z., Xie W., Ashraf U., Li Y., Ma L., Gui R., Pan S., Tian H., Duan M., Wang S. (2020). Exogenous γ-Aminobutyric Acid (GABA) Application at Different Growth Stages Regulates 2-Acetyl-1-Pyrroline, Yield, Quality and Antioxidant Attributes in Fragrant Rice. J. Plant Interact..

[61] Xie W., Kong L., Ma L., Ashraf U., Pan S., Duan M., Tian H., Wu L., Tang X., Mo Z. (2020). Enhancement of 2-Acetyl-1-Pyrroline (2AP) Concentration, Total Yield, and Quality in Fragrant Rice through Exogenous γ-Aminobutyric Acid (GABA) Application. J. Cereal Sci..

[62] Sheteiwy M.S., Shao H., Qi W., Hamoud Y.A., Shaghaleh H., Khan N.U., Yang R., Tang B. (2019). GABA-Alleviated Oxidative Injury Induced by Salinity, Osmotic Stress and Their Combination by Regulating Cellular and Molecular Signals in Rice. Int. J. Mol. Sci..

[63] Hasan M.M., Alabdallah N.M., Alharbi B.M., Waseem M., Yao G., Liu X.-D., Abd El-Gawad H.G., El-Yazied A.A., Ibrahim M.F.M., Jahan M.S. (2021). GABA: A Key Player in Drought Stress Resistance in Plants. Int. J. Mol. Sci..

[64] Cheng S., Fang Z., Wang C., Cheng X., Huang F., Yan C., Zhou L., Wu X., Li Z., Ren Y. (2023). Modulation of 2-Acetyl-1-Pyrroline (2-AP) Accumulation, Yield Formation and Antioxidant Attributes in Fragrant Rice by Exogenous Methylglyoxal (MG) Application. J. Plant Growth Regul..

[65] Luo H., Duan M., Kong L., He L., Chen Y., Wang Z., Tang X. (2021). The Regulatory Mechanism of 2-Acetyl-1-Pyrroline Biosynthesis in Fragrant Rice (*Oryza sativa* L.) under Different Soil Moisture Contents. Front. Plant Sci..

[66] Gui R.F., Jiang H.L., Ashraf U., Li S.Y., Duan M.Y., Pan S.G., Tian H., Tang X.R., Mo Z.W. (2022). Drought Stress at Flowering Stage Regulates Photosynthesis, Aroma and Grain Yield in Fragrant Rice. Appl. Ecol. Environ. Res..

[67] Dangthaisong P., Sookgul P., Wanchana S., Arikit S., Malumpong C. (2023). Abiotic Stress at the Early Grain Filling Stage Affects Aromatics, Grain Quality and Grain Yield in Thai Fragrant Rice (*Oryza sativa*) Cultivars. Agric. Res..

[68] Dey N., Bhattacharyya T., Bhattacharjee S. (2023). Decoding the Impact of Drought Stress Induced Redox-Metabolic Shift in Flag Leaf during Grain-Filling Stage on Kernel Aroma Quality and Productivity in Some Indigenous Aromatic Rice Cultivars of West Bengal, India. J. Plant Growth Regul..

[69] You J., Hu H., Xiong L. (2012). An Ornithine δ-Aminotransferase Gene OsOAT Confers Drought and Oxidative Stress Tolerance in Rice. Plant Sci..

[70] Hoque T.S., Hossain M.A., Mostofa M.G., Burritt D.J., Fujita M., Tran L.-S.P. (2016). Methylglyoxal: An Emerging Signaling Molecule in Plant Abiotic Stress Responses and Tolerance. Front. Plant Sci..

[71] Li L., Huang Z., Zhang Y., Mu Y., Li Y., Nie L. (2025). Regulation of 2-Acetyl-1-Pyrroline (2-AP) Biosynthesis and Grain Quality in Fragrant Rice under Salt Stress. Field Crops Res..

[72] Imran M., Shafiq S., Ashraf U., Qi J., Mo Z., Tang X. (2023). Biosynthesis of 2-Acetyl-1-Pyrroline in Fragrant Rice: Recent Insights into Agro-Management, Environmental Factors, and Functional Genomics. J. Agric. Food Chem..

[73] Fitzgerald T.L., Waters D.L.E., Henry R.J. (2008). The Effect of Salt on Betaine Aldehyde Dehydrogenase Transcript Levels and 2-Acetyl-1-Pyrroline Concentration in Fragrant and Non-Fragrant Rice (*Oryza sativa*). Plant Sci..

[74] Prodhan Z.H., Islam S.A., Alam M.S., Li S., Jiang M., Tan Y., Shu Q. (2022). Impact of *OsBadh2* Mutations on Salt Stress Response in Rice. Plants.

[75] Min M.-H., Maung T.Z., Cao Y., Phitaktansakul R., Lee G.-S., Chu S.-H., Kim K.-W., Park Y.-J. (2021). Haplotype Analysis of BADH1 by Next-Generation Sequencing Reveals Association with Salt Tolerance in Rice during Domestication. Int. J. Mol. Sci..

[76] Zhang H., Xiao Y., Gu X., Chen M., Feng D., Jiang S. (2025). Adaptation of Fragrant Rice in Central China to Climate Change: The Effects of Shifting Sowing Date on Yield and 2-Acetyl-1-Pyrroline Content. Food Energy Secur..

[77] Luo H., Zhang Q., Lai R., Zhang S., Yi W., Tang X. (2024). Regulation of 2-Acetyl-1-Pyrroline Content in Fragrant Rice under Different Temperatures at the Grain-Filling Stage. J. Agric. Food Chem..

[78] Zhang L., Shen C., Zhu S., Ren N., Chen K., Xu J. (2022). Effects of Sowing Date and Nitrogen (N) Application Rate on Grain Yield, Nitrogen Use Efficiency and 2-Acetyl-1-Pyrroline Formation in Fragrant Rice. Agronomy.

[79] Liu Y., Xiao N., Tang D., Li C., Liu X., Xiao F., Xia T. (2025). Transgenic Rice with Microbial High-temperature-resistant Β-glucosidase Gene Significantly Improved 2-acetyl-1-pyrroline Content and Edible Quality. J. Sci. Food Agric..

[80] Luo H., Wu B., Amin B., Li J., Chen Z., Shi J., Huang W., Fang Z. (2025). Amino Acid Regulation in Rice: Integrated Mechanisms and Agricultural Applications. Rice.

[81] Satrio R.D., Fendiyanto M.H., Miftahudin M. (2024). Tools and Techniques Used at Global Scale through Genomics, Transcriptomics, Proteomics, and Metabolomics to Investigate Plant Stress Responses at the Molecular Level. Molecular Dynamics of Plant Stress and Its Management.

[82] Lesk C., Anderson W., Rigden A., Coast O., Jägermeyr J., McDermid S., Davis K.F., Konar M. (2022). Compound Heat and Moisture Extreme Impacts on Global Crop Yields under Climate Change. Nat. Rev. Earth Environ..

[83] Mo Z., Li W., Pan S., Fitzgerald T.L., Xiao F., Tang Y., Wang Y., Duan M., Tian H., Tang X. (2015). Shading during the Grain Filling Period Increases 2-Acetyl-1-Pyrroline Content in Fragrant Rice. Rice.

[84] Siangdee S., Khunpetch S., Promprao S., Sintupachee S. (2024). Enhanced Gene Expression in the Biosynthetic Pathway of 2-Acetyl-1-Pyrroline in “Hom Bon” Fragrant Native Rice in Response to Varied Light Wavelength Conditions. Trends Sci..

[85] Xie H., Xie W., Pan S., Liu X., Tian H., Duan M., Wang S., Tang X., Mo Z. (2021). Effects of Light Quality Treatments during the Grain Filling Period on Yield, Quality, and Fragrance in Fragrant Rice. Agronomy.

[86] Li Y., Liang L., Fu X., Gao Z., Liu H., Tan J., Potcho M.P., Pan S., Tian H., Duan M. (2020). Light and Water Treatment during the Early Grain Filling Stage Regulates Yield and Aroma Formation in Aromatic Rice. Sci. Rep..

[87] Lingaraj V.K., Chakravarthy A.K., Patil S.U. (2015). Impact of Gall Midge, Orseolia Oryzae (Wood-Mason) Infestation on Total Phenols, Proline and Indole Acetic Acid in Paddy (*Oryza sativa* Linn.) Genotypes. New Horizons in Insect Science: Towards Sustainable Pest Management.

[88] Jan R., Asif S., Asaf S., Lubna, Khan Z., Khan W., Kim K.-M. (2024). Gamma-Aminobutyric Acid Treatment Promotes Resistance against *Sogatella furcifera* in Rice. Front. Plant Sci..

[89] Bown A.W., MacGregor K.B., Shelp B.J. (2006). Gamma-Aminobutyrate: Defense against Invertebrate Pests?. Trends Plant Sci..

[90] Bartels D., Sunkar R. (2005). Drought and Salt Tolerance in Plants. Crit. Rev. Plant Sci..

[91] Lin Y.-J., Feng Y.-X., Zhang Q., Yu X.-Z. (2022). Proline-Mediated Modulation on DNA Repair Pathway in Rice Seedlings under Chromium Stress by Integrating Gene Chip and Co-Expression Network Analysis. Ecotoxicology.

[92] Kaur C., Singla-Pareek S.L., Sopory S.K. (2014). Glyoxalase and Methylglyoxal as Biomarkers for Plant Stress Tolerance. Crit. Rev. Plant Sci..

[93] Feng H., Jiang H., Wang M., Tang X., Duan M., Pan S., Tian H., Wang S., Mo Z. (2019). Morphophysiological Responses of Different Scented Rice Varieties to High Temperature at Seedling Stage. Chin. J. Rice Sci..

[94] Prodhan Z.H., Faruq G., Rashid K.A., Taha R.M. (2017). Effects of Temperature on Volatile Profile and Aroma Quality in Rice. Int. J. Agric. Biol..

[95] Kongpun A., Pusadee T., Jaksomsak P., Chinachanta K., Tuiwong P., Chan-In P., Konsaeng S., Pathom-Aree W., Utasee S., Wangkaew B. (2024). Abiotic and Biotic Factors Controlling Grain Aroma along Value Chain of Fragrant Rice: A Review. Rice Sci..

[96] Cebolla-Cornejo J., Roselló S., Valcárcel M., Serrano E., Beltrán J., Nuez F. (2011). Evaluation of Genotype and Environment Effects on Taste and Aroma Flavor Components of Spanish Fresh Tomato Varieties. J. Agric. Food Chem..

[97] Huang A., Sang Y., Sun W., Fu Y., Yang Z. (2016). Transcriptomic Analysis of Responses to Imbalanced Carbon: Nitrogen Availabilities in Rice Seedlings. PLoS ONE.

[98] Fuentes-Peñailillo F., Gutter K., Vega R., Silva G.C. (2024). Transformative Technologies in Digital Agriculture: Leveraging Internet of Things, Remote Sensing, and Artificial Intelligence for Smart Crop Management. J. Sens. Actuator Netw..

[99] Patel A., Shukla C., Trivedi A., Balasaheb K.S., Sinha M.K. (2025). Smart Farming: Utilization of Robotics, Drones, Remote Sensing, GIS, AI, and IoT Tools in Agricultural Operations and Water Management. Integrated Land and Water Resource Management for Sustainable Agriculture.

[100] Mishra H., Mishra D. (2024). AI for Data-Driven Decision-Making in Smart Agriculture: From Field to Farm Management. Artificial Intelligence Techniques in Smart Agriculture.

[101] Ma X., Wang H., Yan S., Zhou C., Zhou K., Zhang Q., Li M., Yang Y., Li D., Song P. (2025). Large-Scale Genomic and Phenomic Analyses of Modern Cultivars Empower Future Rice Breeding Design. Mol. Plant.

[102] Song B., Ning W., Wei D., Jiang M., Zhu K., Wang X., Edwards D., Odeny D.A., Cheng S. (2023). Plant Genome Resequencing and Population Genomics: Current Status and Future Prospects. Mol. Plant.

[103] Huang W. (2024). The Current Situation and Future of Using GWAS Strategies to Accelerate the Improvement of Crop Stress Resistance Traits. Mol. Plant Breed..

[104] Mondal R., Kumar A., Gnanesh B.N. (2023). Crop Germplasm: Current Challenges, Physiological-Molecular Perspective, and Advance Strategies towards Development of Climate-Resilient Crops. Heliyon.

[105] Imran M., Shafiq S., Tang X. (2023). CRISPR-Cas9-mediated Editing of BADH2 Gene Triggered Fragrance Revolution in Rice. Physiol. Plant..

[106] Liao Y., Li M., Wu H., Liao Y., Xin J., Yuan X., Li Y., Wei A., Zou X., Guo D. (2024). Generation of Aroma in Three-line Hybrid Rice through CRISPR/Cas9 Editing of BETAINE ALDEHYDE DEHYDROGENASE2 (OsBADH2). Physiol. Plant..

